# Osteocytes and Primary Cilia

**DOI:** 10.1007/s11914-023-00819-1

**Published:** 2023-09-08

**Authors:** Stefaan W. Verbruggen, Anuphan Sittichokechaiwut, Gwendolen C. Reilly

**Affiliations:** 1 Department of Mechanical Engineering, University of Sheffield, Sheffield, UK; 2 INSIGNEO Institute for in silico Medicine, University of Sheffield, Sheffield, UK; 3 Centre for Predictive in vitro Models, Centre for Bioengineering, School of Engineering and Materials Science, Queen Mary University of London, London, E1 4NS UK; 4 Department of Preventive Dentistry, Faculty of Dentistry, Naresuan University, Phitsanulok, Thailand; 5 Center of Excellence in Biomaterials, Naresuan University, Phitsanulok, Thailand; 6 Kroto Research Institute, Department of Materials Science and Engineering, University of Sheffield, Sheffield, UK

**Keywords:** Bone, Biomechanics, Mechanobiology, Osteocyte, Primary cilium

## Abstract

**Purpose of Review:**

The purpose of this review is to provide a background on osteocytes and the primary cilium, discussing the role it plays in osteocyte mechanosensing.

**Recent Findings:**

Osteocytes are thought to be the primary mechanosensing cells in bone tissue, regulating bone adaptation in response to exercise, with the primary cilium suggested to be a key mechanosensing mechanism in bone. More recent work has suggested that, rather than being direct mechanosensors themselves, primary cilia in bone may instead form a key chemo-signalling nexus for processing mechanoregulated signalling pathways. Recent evidence suggests that pharmacologically induced lengthening of the primary cilium in osteocytes may potentiate greater mechanotransduction, rather than greater mechanosensing.

**Summary:**

While more research is required to delineate the specific osteocyte mechanobiological molecular mechanisms governed by the primary cilium, it is clear from the literature that the primary cilium has significant potential as a therapeutic target to treat mechanoregulated bone diseases, such as osteoporosis.

## Introduction

Osteocytes are by far the most abundant cell type in bone, numbering more than 90% of the bone cell population [1], but are perhaps the most difficult to study given their microenvironment entombed within bone matrix. While much early research into the regulation of bone modelling and remodelling focussed on bone-forming osteoblasts and bone-resorbing osteoclasts, osteocytes have received less attention. Over the past decade or so, the key role of osteocytes in regulating the recruitment and behaviour of other bone cells has been highlighted, particularly in response to mechanical stimulation or exercise [2]. Thus, an ever-increasing body of evidence now supports the paradigm that the osteocyte is the principal cell type responsible for integrating the mechanical and chemical signals that govern modelling and remodelling, and is key to initiating both bone resorption and formation.

Key to this role is the ability of osteocytes to sense mechanical stimulation, integrate these external signals, and transduce them into a biochemical response [1]. For this, the osteocyte is thought to rely on a number of important mechanosensing molecular mechanisms or mechanosensors [1]. The primary cilium has emerged as a key organelle within osteocytes and is known to play a role in the mechanosensitivity of osteocytes and in the adaptive response of bone to mechanical loading [3]. However, whether the primary cilium is a mechanosensor in its own right, or an important integrating microdomain for mechanotransduction, remains debated.

This review begins with a primer on the osteocyte and its major functions within bone tissue, with an emphasis on its role as the key mechanosensing cell within bone tissue. This is then followed by a review of the primary cilium, its biology, and its link with human diseases. The article then explores the putative role for the osteocyte primary cilium in bone mechanosensing, discussing the state-of-the-art, before concluding with a future perspective on the potential of the primary cilium as a therapeutic target in diseases such as osteoporosis.

## Osteocytes

Osteocytes are distributed abundantly and represent the terminal differentiation of the mesenchymal derived osteogenic lineage [4]. This occurs as former osteoblasts become surrounded by unmineralised matrix (osteoid) during bone formation, the result of a complex phenotypic transition. This transition comprises a remarkable shift in both form and function, from a cuboidal morphology organised to secrete extracellular matrix to a dendritic cell with a much reduced cell body and various long, slender processes that connect to their neighbours—both nearby osteocytes and cells on the surface of bone, i.e. osteoblasts and bone lining cells [5]. This stellate morphology results in a distinctive network of interconnected lacunae (cavities containing osteocyte cell bodies) and canaliculi (channels in bone matrix that envelop the osteocyte processes), with this network a defining feature of bone tissue. This unique network of dendritic processes allows contact and communication with other osteocytes via gap junctions and paracrine signalling [6]. These canaliculi are believed to provide a vital system for nutrient supply and waste disposal to the cells and allow transduction of biochemical signals to other cells, both in the matrix and on the bone surface. Osteocytes generate a large number of these canaliculi, numbering between 30 and 50 canaliculi per individual cell [7]. In fact, the network generated by osteocytes is so vast that average canalicular density in the bone of middle-aged women has been calculated as 0.074 ± 0.015 μm/μm^3^ [8], the astonishing equivalent of 74 km/cm^3^ [9]. So crucial is this network that disruption of it in old bone, with a loss of canaliculi and changes in lacunar geometries [10–12], is thought to underly the decreased mechanosensitivity observed with aging [13]. A mesh-like glycocalyx, or pericellular matrix (PCM), surrounds the osteocyte and is thought to tether it to the extracellular matrix (ECM), while punctate integrin attachments between the cell processes and the matrix have been identified in the canaliculi [14]. It has been proposed that both of these extracellular attachments may act to amplify strain signals to the osteocyte through their connections with the cytoskeleton [15], and experimental studies have shown that osteocyte cell processes are significantly more mechanosensitive than the cell bodies [16].

The osteocyte processes themselves form before the bone matrix mineralises [5] and, while the mechanisms underlying this process are not fully understood, they appear to be dynamically regulated rather than random. Osteocytes in vitro, for example, can elongate their cell processes by secreting E11/gp38 dendritic proteins in response to fluid shear stress [17]. Matrix metalloproteinases (MMPs) are also required to complete the extension of processes through bone matrix in vivo [17]. Holmbeck and colleagues showed that transgenic mice lacking membrane type matrix metalloproteinase 1 (MT1-MMP or MMP-14) form osteocyte processes but never develop pericellular space around them [18]. Inoue et al. also demonstrated that MMP-2 is essential for the formation of osteocyte canaliculi and networks in calvarial bone [19].

Upon terminal differentiation and mineralisation of their surrounding osteoid, osteocytes are entombed until they die, often living for years in situ until the bone in which they are located is replaced with new bone via remodelling. In extreme examples of bones that do not remodel, such as the bones of the inner ear, evidence suggests that even in 95-year-old humans the osteocytes present are as old as the individual [20].

### Osteocytes as Mechanosensors

**Fig. 1:**
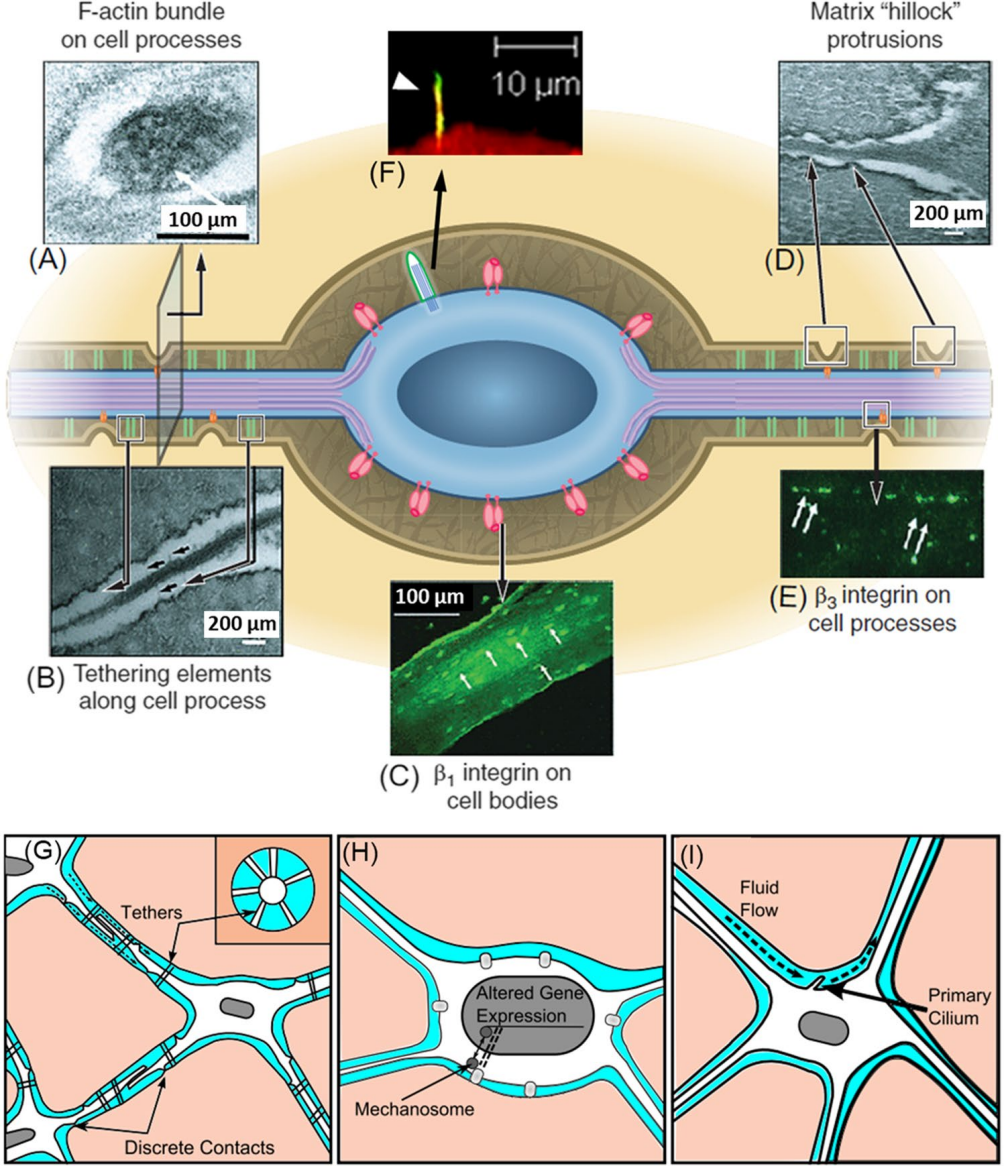
Osteocytes sense mechanical stimulation in vivo, with a number of potential sensing mechanisms identified (black and white arrows indicate mechanosensors): (**A**) TEM image of an osteocyte process displaying the actin cytoskeleton; (**B**) TEM image of proteoglycan pericellular matrix (PCM) tethering elements (black arrows) bridging an osteocyte cell process to the bony canalicular wall; (**C**) fluorescent immunohistochemical staining showing that β1 integrins (white arrows) are located only on osteocyte cell bodies; (**D**) TEM image demonstrating the discrete extracellular matrix (ECM) projections from the canalicular wall that contact osteocyte processes (black arrows); (**E**) fluorescent immunohistochemical staining for β3 integrins (white arrows) that are present in a punctate pattern along osteocyte processes, with similar periodicity and spacing pattern to ECM projections; and (**F**) the primary cilium (white arrow) on the osteocyte cell body. These sensing mechanisms may experience either interstitial fluid flow or strain of the surrounding matrix: (**G**) combined fluid shear and matrix strain via tethering elements or focal adhesions along the dendritic cell processes; (**H**) matrix strain via focal adhesions on the cell body; and (**I**) direct fluid flow sensing via the primary cilium in the lacunar cavity. Adapted from Schaffler et al. [1], Verbruggen and McNamara [21], and Duffy and Jacobs [22]

Bone has long been recognised as a most mechanosensitive tissue, with research theories in this field stretching back to those of Julius Wolff in the 1800s. The basic rule of adaptation of bone structure in response to mechanical loading is, to this day, commonly referred to as Wolff’s Law, with further work carried out in orthodontics by his contemporary Rudolf Virchow. Yet, despite this well-established paradigm, the precise mechanism through which this mechanical stimulation is perceived and transduced into a biological outcome has eluded researchers ever since. While osteocytes were gradually accepted to be the key sensors of mechanical loading in bone tissue, these complex cells and their intricate environment have presented a bewildering array of experimental challenges, molecular sensors, and signalling pathways that have hampered attempts to pinpoint the mechanism underpinning their mechanosensitivity (Fig. 1).

In the unique mechanical environment of the lacuno-canalicular network, osteocytes experience a range of mechanical stimuli, including substrate deformation, fluid shear, or changes in hydrostatic pressure; and osteocytes experience all of these stimuli to some degree. During normal physiological loading (e.g. walking), interstitial fluid pressure causes flow into and out of the lacuno-canalicular network and produces shear stresses along osteocyte membranes [23]. Changes in interstitial hydrostatic pressure also result [24], as does deformation of bone matrix itself [25], though the rigidity of the tissue indicates that such deformations are very small [26]. Physiological tissue strain experienced by bone ranges from 400 to 3000 microstrain (με, 0.04–0.3%) [26], with the vast majority of in vivo loading occurring at the low end of this range [26]. However, mechanical strains that elicit biological responses from osteocytes in vitro range from 5000 to 10,000 με (5–10%) [27], similar to all connective tissue cells in vitro. Moreover, whole bone tissue typically breaks at approximately 1% strain [27]. Thus, it was thought to be unrealistic for healthy osteocytes to be experiencing such high strains in vivo. These findings implied that osteocytes must experience something very different from mechanical strain at the cellular scale in situ. A theory of strain amplification due to the local geometries and micro-architecture around osteocytes was developed over time by Weinbaum, Schaffler, and others [15, 28–30], with direct experimental evidence of amplification of loads to stimulatory strain levels shown in individual osteocytes in situ [31]. However, it is important to note that the observed strains in this study may themselves have been induced by fluid flow.

Therefore, the broad sweep of evidence has led to the general consensus that mechanical loading-induced fluid flow in the lacuno-canalicular network is likely the predominant force that osteocytes recognise and respond to by regulating bone modelling and/or remodelling activity [27]. However, exactly how this fluid flow is sensed remains a topic of debate, with a number of different mechanisms of action proposed. This review will focus on perhaps the most hotly debated of these putative osteocyte mechanosensors, the primary cilium.

## The Primary Cilium

The primary cilium is a solitary, immotile organelle that protrudes from the cell surface of almost all mammalian cells. Cilia in general were discovered with the advent of microbiology, first described in protozoa by the Dutch lens maker and founder of the field, Antony van Leeuwenhoek, more than 300 years ago. Primary cilia were first observed in mammalian cells by Swiss anatomist Karl Zimmermann in 1898 [32]. Noting their presence in several cell types from tissues as diverse as the kidney and pancreas, Zimmerman presciently hypothesised a sensory function for these cilia. This hypothesis was neglected for more than a century, with the primary cilium thought to be a vestigial organelle of negligible importance in human biology or medicine [33]. The past couple of decades have turned this thinking on its head, with renewed interest and numerous studies demonstrating its importance to basic cell structures and functions. In this review, we will focus on the body of evidence implicating the primary cilium in bone biology and disease, shedding particular light on recent advances in our understanding of the osteocyte primary cilium. We first give a brief overview of primary cilium biology, along with a discussion of observed ciliopathies. We will then discuss recent advances in primary cilia research in bone, focusing on proposed roles of osteocyte primary cilia in mechanobiology and potential related signalling pathways. We conclude by exploring future areas of research and unanswered questions.

### Primary Cilium Biology

**Fig. 2:**
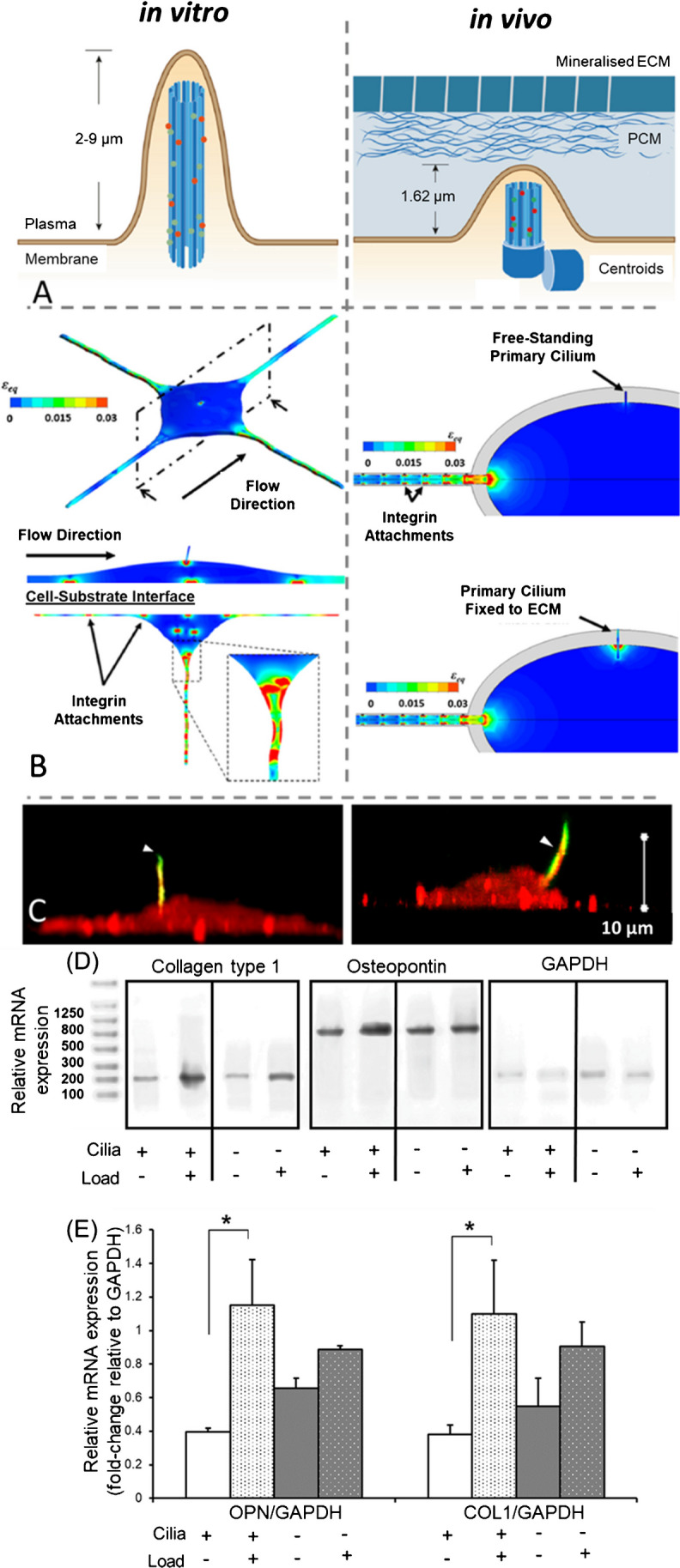
(**A**) Configuration of the osteocyte primary cilium when cultured in vitro, or when in situ in its lacuna in vivo. Adapted from Qin et al. [36]. (**B**) Computational models predict that high strain is likely experienced by both integrin attachments and the primary cilium under in vitro flow conditions. However, while integrin attachments were similarly stimulated under in vivo flow, the primary cilium was only sufficiently mechanically stimulated if connected to the surrounding matrix. Adapted from Vaughan et al. [37]. **(C)** Evidence of hyaluronic acid (red), a key component of the cell glycocalyx and the pericellular matrix, present on the entire cell surface of MLO-A5 osteoblastic cells in vitro, an co-localising along the length of the primary cilium (acetylated α-tubulin, green) [38]. Mechanical loading upregulates collagen type I and osteopontin by MLO-A5 osteoblastic cells, with this effect blocked by pre-treatment with chloral-hydrate to disrupt primary cilia, as measured by relative gene expression via RT-PCR, shown as **(D)** gels and **(E)** fold-change compared to their controls as measured by band density relative to GAPDH. (*p < 0.05, Tukey’s post hoc pairwise comparison, *n* = 3)

The primary cilium is a microtubule-based structure, constructed to protrude from the cell body. It is similar in structure to motile cilia and flagella in eukaryotic cells, as all three comprise an axoneme of nine microtubule doublets extending from the basal body into the extracellular space. A key common feature is the microtubule doublets, which serve as the basis for structure and rigidity in these organelles. In contrast to the motile cilium and flagellum, the primary cilium lacks two central microtubules, resulting in a 9 + 0 arrangement (Fig. 2A) [34]. The primary cilium also lacks other axonemal components, including radial spokes, dynein arms, and nexin links [35]. Motile cilia display an order of magnitude greater flexural rigidity than primary cilia, and it is likely these missing components that reinforce the axoneme and provide this extra stiffness [3].

The formation and maintenance of the primary cilium occur in the G1 or G0 phase of the cell cycle, through a process called intraflagellar transport (IFT). As primary cilia do not contain protein-forming cellular machinery themselves, all constituent ciliary proteins are formed elsewhere in the cell and transported to the cilium through IFT. IFT comprises a bidirectional trafficking system that transports proteins along the ciliary axoneme, with kinesin-2 responsible for anterograde movement and dynein serving as the retrograde motor. Kif3a and polaris, two proteins previously identified for their implications in polycystic kidney disease, are now known to play key roles in IFT [39–41]. The anterograde motor kinesin-2 is formed by Kif3a and Kif3b proteins [41]. Polaris forms a critical component of a multi-protein IFT complex used in carrying other proteins [39, 40], and is now also known as Ift88.

As mentioned briefly above, while primary cilia are found on nearly all human cell types, with the exception of those of myeloid and lymphoid lineages [3], they are not a constant presence on these cells. Instead, the occurrence of primary cilia is cell cycle-dependent and the result of a dynamic process of assembly and resorption. Assembly of the cilium occurs in the interphase with the most cilia found on non-proliferating G0–G1 cells [3]. The mother centriole converts into the basal body, providing an anchor from which IFT and kinesin-2 can extend the primary cilium length. Disassembly and resorption of the primary cilium occur prior to mitosis and re-entry into the cell cycle.

The primary cilium maintains this structure and also fulfils many of these functions in the biology of bone cells. As we will discuss, evidence has emerged, including in the form of cilia-related conditions, that imply additional roles for the primary cilium in bone tissue and bone diseases.

### Ciliopathies

A large number of disorders have implicated defects in primary cilia structure and function as key mediators, with conditions related to these defects referred to as ciliopathies. As the primary cilium is ubiquitous in mammalian cells, defects regularly present clinically as multisystemic dysfunction [3]. Common clinical hallmarks of ciliopathies include renal disease, retinal degeneration, and cerebral anomalies [42]. Renal disease in particular is highly linked to primary cilia defects and has been studied in detail, with the first hint of a link between primary cilia and polycystic kidney disease observed by Murcia et al. in 2000 [43]. Using the Oak Ridge Polycystic Kidney mouse model for polycystic kidney disease, the authors found mutations in the Tg737 gene led to left–right axis defects and primary cilia defects [43]. Given the importance of the protein encoded by Tg737 for polarity defects in the positioning of the associated receptors and to the left–right polarity defects in the early embryo, the study authors named the protein ‘polaris’, with pleasing connotations as a star guiding development [43]. Later, other ciliary proteins in addition to polaris, such as Kif3a, were also associated with polycystic kidney disease [44]. Interestingly, proteins such as polycystin-1 and polycystin-2, which are linked to polycystic kidney disease, have been found to localise to the primary cilium [45]. Though much less well studied, a common feature of ciliopathies is a range of varied skeletal abnormalities, and it has recently been shown that the bone cells of polycystic kidney disease and chronic kidney disease patients display abnormal primary cilia [46]. The link between these findings and skeletal abnormalities is possibly due to the role of primary cilia in controlling cell alignment and patterning during bone development [47].

The most common skeletal abnormalities observed with ciliopathies include congenital defects of the extremities, craniofacial defects, and ossification disorders. The ciliopathies underlying Joubert syndrome [48], Mainzer-Saldino syndrome [49, 50], and polycystic kidney disease [51] are all known to affect the extremities of the body, with abnormal structure or physiology observed in the hands and feet. Simpson Golabi Behmel syndrome [52] and Oral-Facial-Digital syndrome [53, 54] exhibit craniofacial defects alongside the extremities, while patients with ciliopathies known as asphyxiating thoracic dystrophy [55], Ellis-van Creveld syndrome [56], and nephronophthisis [57, 58] present with abnormal stature or skeletal dysplasia in addition to extremity defects. Meckel-Gruber syndrome [59] is particularly severe and includes all of the above.

A final set of ciliopathies are known to additionally cause spinal abnormalities, including scoliosis and kyphosis. Senior-Loken syndrome [60] includes this presentation along with extremities and skeletal dysplasia defects, while in Bardet-Biedl syndrome [61, 62] the spine, skull, and extremities are most affected. The most complex ciliopathy known to affect the skeleton is Alström syndrome [63], with shortened stature and abnormalities of the spine, skull, and extremities.

### Mechanosensors or Mechanosignalling Mediators?

It took more than a century after the primary cilium’s sensory function was first hypothesised before its mechanosensory function was clearly defined [3]. It was first shown in kidney cells that the primary cilium bends under fluid flow [64], with this bending then being modelled to propose a role as a flow sensor in the kidney [35]. This idea was subsequently supported by experimental evidence, showing that primary cilia could sense fluid flow and that this mechanosensation formed part of a calcium signalling system [65]. This led to the development of the field of primary cilium mechanobiology, and the idea that it may present a novel and widespread therapeutic target in cellular mechanotransduction.

The primary cilia of osteoblasts and osteocytes were first reported in 1972 [66], but did not receive significant attention until 2003 when Whitfield proposed that the solitary cilium could be a mechanosensing organelle in bone by sensing flow of interstitial lacuno-canalicular fluid [67]. While less evidence for a mechanosensory role for primary cilia is available in bone [68] and they appear to have low expression in the bone marrow [69], since the bending of a kidney cell’s primary cilium enables the cell to sense fluid flow, the bending of a primary cilium of a bone cell by moving extracellular fluid is thought to behave similarly [67, 70]. This led to the emerging role of the primary cilium as an area of interest for bone [71] and biomechanics [72, 73] researchers. Early evidence suggested the importance of the primary cilium in regulating mechanotransduction in human bone MSCs, indicating primary cilium-mediated mechanoregulation of osteogenic fate [74]. It was later observed that specific mechanosensing molecular mechanisms localise to the primary cilium, with TRPV4-mediated mechanotransduction partly reliant on the presence of a cilium [75], while general cilium-regulated mechanotransduction by MSCs is reliant on a novel mechanosensitive G-coupled protein receptor (Gpr161) [76]. While this promising research focused earlier in the osteogenic lineage, evidence also suggested that paracrine signalling between the primary mechanosensory cell, the osteocyte, was also governed by the primary cilium [77].

Using mechanical theory to follow this logic, studies have modelled the bending of the osteocyte primary cilium under applied fluid flow, using both analytical models [78] and finite element (FE) modelling [79]. A more recent study has combined FE with computational fluid dynamics (CFD) to generate an advanced in silico model of the flow around osteocytes and the deformation this would induce in the cells [37]. Crucially, this study modelled both in vitro and in vivo conditions, and compared the primary cilium with other mechanosensors, such as integrin attachments between the surface of the cell and the ECM [37]. Under in vitro fluid flow stimulation, both integrin attachments and primary cilia were found to be highly stimulated (Fig. 2B) [37]. While integrin attachments resulted in similarly high amounts of strain transfer under in vivo flow conditions, the primary cilium was only sufficiently stimulated if partly attached to the surrounding extracellular matrix [37]. Therefore, while primary cilia likely play a role in mediating bone mechanotransduction in vitro, it remains to be seen whether a typical primary cilium could undergo sufficient strain in vivo to directly sense mechanical loads [37]. Interestingly, chondrocytes have been shown to express a number of α and β integrins on their primary cilia, alongside other ECM receptors [80], indicating that the osteocyte primary cilium may have a similar capability to attach directly to the lacunar wall in this manner. Similarly, primary cilia of MLO-A5 osteoblastic cells have been shown to produce hyaluronic acid when cultured in vitro (Fig. 2C) [38]. Hyaluronic acid is a critical component of the cell glycocalyx and is present in the osteocyte PCM. Given evidence that degradation of hyaluronic acid results in destruction of the glycocalyx and a reduction in mechanosensitivity in osteocytes in vitro [81], this may provide a potential molecular mechanism by which the osteocyte primary cilium is mechanically stimulated in vivo.

Testing the relationship between osteocyte primary cilia and bone remodelling, two in vivo studies applied a conditional knockout of kif3a using a Cre-lox system (global knockouts are embryonically lethal [82]) and found lower rates of bone formation compared to wild type. By breeding a Cola1(I)2.3-Cre, Temiyasathit et al. deleted kif3a expression in osteoblasts. While Colα1(I)2.3 is not expressed in osteocytes, this ablation is carried through as the osteoblasts mature [83]. No skeletal abnormalities were observed in young or mature mice, in either control or knockouts [83]. However, when mechanical loading was applied in the form of cyclic axial compression of the forelimb, control mice produced significantly more new bone than the knockouts [83]. This implies a mechanosensory or intermediary role for the primary cilium or the kif3a pathway in bone, though whether via osteoblast or osteocyte activity was unclear.

A similar study attempted to differentiate between osteoblast and osteocyte effects by instead tying the kif3a knockout to osteocalcin, which is expressed in late-stage osteoblasts and in osteocytes [84]. Osteopoenia was observed in peri-adolescent knockout mice but not in controls, and osteogenic gene expression in tibiae was significantly downregulated [84]. The authors isolated and immortalised osteoblasts from these mice, and found a less osteoblastic differentiation and an abrogated calcium response to fluid flow [84]. This again implies a disruption of mechanosensing via this primary cilium pathway for skeletal development, though it remains unclear through which cell type. Added to this, recent unpublished work by ourselves has demonstrated that the primary cilium may mediate the matrix-forming response of bone cells to mechanical loading when grown in 3D cultures typical of tissue-engineering protocols (Fig. 2D, E). Mechanical loading of MLO-A5 osteoblastic cells cultured in 3D polyurethane scaffolds led to upregulation of COL1 and OPN mRNA expression, whereas pre-treatment with chloral-hydrate, a chemical that disrupts microtubule assembly, inhibited the mechanobiological response (Fig. 2D, E).

Further evidence for the mechanosensory role of primary cilia in osteocytes arises from work investigating the localisation of proteins to the primary cilium, which itself forms a unique microdomain within the cell to which proteins are trafficked [85]. Examples of these include mechanosensitive ion channels such as transient receptor potential vanilloid 4 (TRPV4) and the polycystin 1/2 (PC1/2) ion channel complex [85]. At present, the exact role of primary cilia in downstream mechanotransduction cascades, and whether signalling arises directly as a result of ciliary bending in response to fluid shear, is much debated. Disruption of cilia-localised proteins has been shown by a number of groups to diminish cell mechanotransduction, as we will now discuss. Knockdown of osteocyte primary cilia was recently shown to downregulate receptors for parathyroid hormone (PTH)-related protein, a known mechanosensitive signalling molecule, via a Gli-1-dependent mechanism [86]. Abrogating the polycystin channel in osteocytes and osteoblasts reduces load-induced bone formation, while knockdown of TRPV4 in osteocytes abrogates fluid flow-induced cyclooxygenase-2 expression [85, 87, 88]. As well as these mechanosensitive ion channel proteins, studies have investigated disruption of adenylyl cyclases, which are required to convert adenosine triphosphate (ATP) to the important second messenger cyclic adenosine monophosphate (cAMP) [89]. One particular adenyl cyclase, AC6, is known to localise to primary cilia in osteocytes [89], knockdown of which in vitro disrupts osteocyte mechanosensing [89]. A global knockout in vivo, as well as knock-outs targeted to bone MSCs [90], attenuates whole-bone adaptation in response to load [85, 89]. Furthermore, knockdown of another adenyl cyclase that localises to the primary cilium in osteocytes, AC3, was recently shown in vitro to moderate both primary cilium length and osteocyte mechanotransduction, presenting a new therapeutic avenue for bone disease treatments [91].

A particular question in the field has been whether increased primary cilium length causes increased ciliary membrane strain under mechanical stimulation, inducing transduction though stretch-activated ion channels [92]. A counter-argument is that increased primary cilium length would likely be coupled with increased production of ciliary proteins and thus inherently potentiates greater mechanotransduction signalling. Indeed, the osteocyte primary cilium has been shown to be a key mediator in response to a range of sensing mechanisms, including pulsed electro-magnetic fields [93, 94] and simulated microgravity [95]. This research problem has been made more tractable by evidence suggesting that primary cilia length can be pharmacologically manipulated to control cellular mechanotransduction, as we will now discuss.

An example of pharmacological lengthening is fenoldopam, an agonist for dopamine-like 1 receptor that localises to the primary cilium, treatment with which increases primary cilia length in endothelial cells, kidney epithelial cells, and osteocytes [92, 96, 97]. When delivered as an intravenous injection fenoldopam is known to lower blood pressure in hypertensive patients and mice, an effect produced by increasing the primary cilia-mediated flow-induced nitric oxide production [96]. It has been shown that treatment of osteocytes with fenoldopam significantly increases primary cilia length while enhancing osteocyte mechanosensitivity and osteogenic signalling [92]. Conversely, pharmacological intervention by treatment with tubastatin, an HDAC6-specific deacetylase inhibitor that causes an increase in primary-cilia microtubule acetylation, both stabilises the primary cilium and increases its stiffness. By making the primary cilium more resistant to fluid flow deformation in this manner, the Jacobs group demonstrated reduced osteogenic signalling in vitro [98]. Additionally, in vitro testing using a range of pharmacological treatments (chloral hydrate, Gd^3+^, Li^+^, rapamycin) to both shorten and lengthen the primary cilium of osteocyte cells indicates a close structure-function relationship between primary cilium length and mechanosensitivity [99].

By manipulating osteocyte primary cilia and ciliary-associated proteins via altered gene expression, we can also direct mechanically induced intercellular communication. Knockdown of a key ciliary structural protein (IFT88) [92], mechanosensitive ion channels (TRPV4 and PC2) [85], and ciliary-localised signal transduction proteins (AC3 [91], AC6 [89]) have all resulted in decreased mechanically stimulated pro-osteogenic paracrine signalling to osteoblasts. Interestingly, it has been shown that TRPV4, and not PC2, is the main ciliary mechanosensitive ion channel over short periods of mechanical stimulation [85]. TRPV4 and PC2, however, may form a heteromeric channel within the cilium, suggesting that their function may in fact be linked, warranting further investigation into their long-term ciliary mechanosensing function [100].

Taken together, the studies discussed in this section suggest there is a clear link between osteocyte primary cilium length and mechanosensitivity, as well as production of mechanosensitive proteins. However, a recent study has shown that changes in ciliary, but not cytosolic, cAMP regulate transcriptional activity via the hedgehog pathway, which is traditionally strongly linked to cilia structure and function [101]. As well as providing further evidence that the primary cilium is a unique therapeutic target, distinct from the rest of the cell, the current literature also suggests that, rather than direct mechanosensation by the cilium, it is increased size of this microdomain, and associated mechanosensory proteins and channels, that is the mechanism of action of these cilia-lengthening interventions.

### Osteocytes and Primary Cilia in Osteoporosis

Despite being one of the most studied bone diseases, precisely how osteoporosis affects bone mechanosensitivity is not well understood. However, disease traits have been linked to changes in osteocyte lacunae in human bone [102], and there now exists a body of evidence that osteoporosis may be, in part, a disease of impaired osteocyte mechanosensitivity [103]. While osteocytes are clearly affected, the impact of osteoporosis on primary cilium function is not yet clear, leading to its proposal as a disease candidate for ciliotherapies. Using ovariectomised models to induce post-menopausal osteoporosis, it has been shown that oestrogen deficiency alone does not affect load-induced bone formation, at 1 week post-surgery [104]. Similarly, ovariectomy has been shown to have no effect on load-induced periosteal bone formation [105], again suggesting that ovariectomy does not alter the mechanosensitivity of bone. However, it has been shown that ovariectomy in rats results in increased lacuno-canalicular porosity and vascular porosity [106]. Computational modelling suggests that this increase in porosity could possibly lead to decreased interstitial fluid velocity and thus decreased shear stress mechanical stimulation [37, 107], but this has not yet been explicitly shown in vivo. Taken together, these findings suggest reduced fluid-induced mechanical stimulation in ovariectomised animals, potentially decreasing the mechanotransduction response of osteocytes. Moreover, when cultured under oestrogen deficiency in vitro, osteocyte response to oscillatory fluid flow stimulation was abrogated [108]. Conversely, it has also been demonstrated experimentally that osteoporotic bone cells actually experience greater strains than osteocytes in healthy bone, due to the loss of bone mass to resist loading, and thus potentially receive greater mechanical stimulation [31].

At present, it is clear that the exact manner through which oestrogen deficiency-induced disruption of bone architecture leads to changes in strain, mechanical stimulation, and mechanotransduction is not fully understood. Irrespective of precise disruption to mechanosensing mechanisms at play during osteoporosis, it is well established that mechanical stimulation can moderate the negative effects of osteoporosis on bone mass [109]. Previous work by the Jacobs group has shown that fenoldopam, a primary cilium lengthening compound, can also increase osteocyte mechanosensitivity in vitro [92]. Furthermore, their results suggest that in animal models, pharmacologically induced lengthening of primary cilia via fenoldopam promotes load-induced bone adaptation in ovariectomised mice [98]. Lastly, by focusing on another bone cell type, osteoclasts, the group showed that loss of primary cilia is required for osteoclastogenesis [110]. Indeed, treatment with the same primary cilium-lengthening agent fenoldopam was capable of reducing osteoclast formation through maintenance of macrophage primary cilia, and therefore may also have a role as an anti-resorptive treatment [110]. However, it has also recently been shown that oestrogen withdrawal from osteocytes in vitro, to mimic osteoporotic conditions, can itself cause primary cilium elongation and results in pro-osteoclastogenic paracrine signalling [111]. Therefore, while further study is still required to unpick this mechanism, manipulation of primary cilia to either sensitise bone to mechanical stimulation, or to inhibit osteoclastogenesis, remains a promising approach to preventing and treating osteoporosis.

## Conclusions

Research over a number of decades has now firmly established the primary cilium, once considered a vestigial organelle, as an important chemosensory and mechanosensory nexus in a range of tissues. Mounting evidence from both in vitro and in vivo studies suggests that the primary cilium of the osteocyte plays a key role in bone mechanobiology, acting at least as an important mediating signalling domain for transduction of mechanical stimuli into biochemical signals. A range of key molecular mechanisms appear to be mediated in this manner: Pkd1, adenylyl cyclases, and Wnt signalling. However, while major steps have been taken in understanding osteocyte primary cilia function and the development of methods to manipulate its structure pharmacologically, whether it acts as a mechanosensor for osteocytes in and of itself remains unclear. Furthermore, whether defects in osteocytes have direct implications for the skeleton as ciliopathies, and whether primary cilia offer intervention pathways in bone diseases such as osteoporosis, requires further investigation.

## Data Availability

Data is available from authors upon request.

## References

[1] Schaffler MB, Cheung W-Y, Majeska R, Kennedy O (2014). Osteocytes: master orchestrators of bone. Calcif Tissue Int.

[2] Birmingham E, Niebur GL, McHugh PE, Shaw G, Barry FP, Mcnamara LM (2012). Osteogenic differentiation of mesenchymal stem cells is regulated by osteocyte and osteoblast cells in a simplified bone niche. Eur Cells Mater.

[3] Nguyen AM, Jacobs CR (2013). Emerging role of primary cilia as mechanosensors in osteocytes. Bone.

[4] Frost HM (1960). In vivo osteocyte death. JBJS.

[5] Palumbo C, Palazzini S, Zaffe D, Marotti G (1990). Osteocyte differentiation in the tibia of newborn rabbit: an ultrastructural study of the formation of cytoplasmic processes. Cells Tissues Organs.

[6] Xia X, Batra N, Shi Q, Bonewald LF, Sprague E, Jiang JX (2010). Prostaglandin promotion of osteocyte gap junction function through transcriptional regulation of connexin 43 by glycogen synthase kinase 3/β-catenin signaling. Mol Cell Biol.

[7] Buenzli PR, Sims NA (2015). Quantifying the osteocyte network in the human skeleton. Bone.

[8] Repp F, Kollmannsberger P, Roschger A, Kerschnitzki M, Berzlanovich A, Gruber GM, Roschger P, Wagermaier W, Weinkamer R (2017). Spatial heterogeneity in the canalicular density of the osteocyte network in human osteons. Bone Rep.

[9] Dallas SL, Moore DS. Using confocal imaging approaches to understand the structure and function of osteocytes and the lacunocanalicular network. Bone. 2020;138. 10.1016/j.bone.2020.115463. 10.1016/j.bone.2020.115463 PMC7423610 32512167

[10] Heveran CM, Schurman CA, Acevedo C, Livingston EW, Howe D, Schaible EG, Hunt HB, Rauff A, Donnelly E, Carpenter RD (2019). Chronic kidney disease and aging differentially diminish bone material and microarchitecture in C57Bl/6 mice. Bone.

[11] Heveran CM, Rauff A, King KB, Carpenter RD, Ferguson VL (2018). A new open-source tool for measuring 3D osteocyte lacunar geometries from confocal laser scanning microscopy reveals age-related changes to lacunar size and shape in cortical mouse bone. Bone.

[12] Tiede-Lewis LM, Xie Y, Hulbert MA, Campos R, Dallas MR, Dusevich V, Bonewald LF, Dallas SL. Degeneration of the osteocyte network in the C57BL/6 mouse model of aging. Aging (Albany. NY). 2017;9:2190–2208. 10.18632/aging.101308. 10.18632/aging.101308 PMC5680562 29074822

[13] Schurman CA, Verbruggen SW, Alliston T (2021). Degenerated lacunocanalicular networks, mass transport and osteocyte pericellular fluid flow in bone with aging and disrupted TGFB signaling. Proc Natl Acad Sci U S A.

[14] McNamara LM, Majeska RJ, Weinbaum S, Friedrich V, Schaffler MB (2009). Attachment of osteocyte cell processes to the bone matrix. Anat Rec.

[15] Wang, Y.; McNamara, L.M.; Schaffler, M.B.; Weinbaum, S. A model for the role of integrins in flow induced mechanotransduction in osteocytes. Proc Natl Acad Sci. 2007;104:15941 LP–15946. 10.1073/pnas.0707246104. 10.1073/pnas.0707246104 PMC2000405 17895377

[16] Adachi T, Aonuma Y, Tanaka M, Hojo M, Takano-Yamamoto T, Kamioka H (2009). Calcium response in single osteocytes to locally applied mechanical stimulus: differences in cell process and cell body. J Biomech.

[17] Zhang K, Barragan-Adjemian C, Ye L, Kotha S, Dallas M, Lu Y, Zhao S, Harris M, Harris SE, Feng JQ (2006). E11/gp38 selective expression in osteocytes: regulation by mechanical strain and role in dendrite elongation. Mol Cell Biol.

[18] Holmbeck K, Bianco P, Pidoux I, Inoue S, Billinghurst RC, Wu W, Chrysovergis K, Yamada S, Birkedal-Hansen H, Poole AR (2005). The metalloproteinase MT1-MMP is required for normal development and maintenance of osteocyte processes in bone. J Cell Sci.

[19] Inoue K, Mikuni-Takagaki Y, Oikawa K, Itoh T, Inada M, Noguchi T, Park J-S, Onodera T, Krane SM, Noda M (2006). A crucial role for matrix metalloproteinase 2 in osteocytic canalicular formation and bone metabolism. J Biol Chem.

[20] Bloch SL, Kristensen SL, Sørensen MS (2012). The viability of perilabyrinthine osteocytes: a quantitative study using bulk-stained undecalcified human temporal bones. Anat Rec Adv Integr Anat Evol Biol.

[21] Verbruggen SW, McNamara LM. Bone mechanobiology in health and disease. Mechanobiol Heal Dis. 2018: 157–214. 10.1016/B978-0-12-812952-4.00006-4.

[22] Duffy MP, Jacobs CR (2015). Seeing the unseen: cell strain and mechanosensing. Biophys J.

[23] Piekarski K, Munro M (1977). Transport mechanism operating between blood supply and osteocytes in long bones. Nature.

[24] Liu C, Zhao Y, Cheung W-Y, Gandhi R, Wang L, You L (2010). Effects of cyclic hydraulic pressure on osteocytes. Bone.

[25] Adachi T, Aonuma Y, Ito S-I, Tanaka M, Hojo M, Takano-Yamamoto T, Kamioka H. Osteocyte calcium signaling response to bone matrix deformation. J Biomech. 2009;42:2507–2512. 10.1016/j.jbiomech.2009.07.006. 10.1016/j.jbiomech.2009.07.006 19665124

[26] Fritton SP, McLeod KJ, Rubin CT (2000). Quantifying the strain history of bone: spatial uniformity and self-similarity of low-magnitude strains. J Biomech.

[27] You J, Yellowley CE, Donahue HJ, Zhang Y, Chen Q, Jacobs CR (2000). Substrate deformation levels associated with routine physical activity are less stimulatory to bone cells relative to loading-induced oscillatory fluid flow. J Biomech Eng.

[28] Nicolella DP, Nicholls AE, Lankford J, Davy DT (2001). Machine vision photogrammetry: a technique for measurement of microstructural strain in cortical bone. J Biomech.

[29] Verbruggen SW, Vaughan TJ, McNamara LM (2012). Strain amplification in bone mechanobiology: a computational investigation of the in vivo mechanics of osteocytes. J R Soc Interface.

[30] Verbruggen SW, Vaughan TJ, McNamara LM (2014). Fluid flow in the osteocyte mechanical environment: a fluid-structure interaction approach. Biomech Model Mechanobiol.

[31] Verbruggen SW, Mc Garrigle MJ, Haugh MG, Voisin MC, McNamara LM (2015). Altered mechanical environment of bone cells in an animal model of short- and long-term osteoporosis. Biophys J.

[32] Zimmermann KW. Beiträge zur Kenntniss einiger drüsen und epithelien. Arch Mikr Anat. 1898;52:552–706.

[33] Federman M, Nichols G (1974). Bone cell cilia: vestigial or functional organelles?. Calcif Tissue Res.

[34] Temiyasathit S, Jacobs CR (2010). Osteocyte primary cilium and its role in bone mechanotransduction. Ann NY Acad Sci.

[35] Schwartz EA, Leonard ML, Bizios R, Bowser SS (1997). Analysis and modeling of the primary cilium bending response to fluid shear. Am J Physiol Physiol.

[36] Qin L, Liu W, Cao H, Xiao G. Molecular mechanosensors in osteocytes. Bone Res. 2020;8. 10.1038/s41413-020-0099-y. 10.1038/s41413-020-0099-y PMC7280204 32550039

[37] Vaughan TJ, Mullen CA, Verbruggen SW, McNamara LM (2015). Bone cell mechanosensation of fluid flow stimulation: a fluid–structure interaction model characterising the role integrin attachments and primary cilia. Biomech Model Mechanobiol.

[38] Delaine-Smith RM, Sittichokechaiwut A, Reilly GC (2014). Primary cilia respond to fluid shear stress and mediate flow-induced calcium deposition in osteoblasts. FASEB J.

[39] Kobayashi T, Dynlacht BD (2011). Regulating the transition from centriole to basal body. J Cell Biol.

[40] Lucker BF, Miller MS, Dziedzic SA, Blackmarr PT, Cole DG (2010). Direct interactions of intraflagellar transport complex B proteins IFT88, IFT52, and IFT46. J Biol Chem.

[41] Praetorius HA, Spring KR (2005). A physiological view of the primary cilium. Annu Rev Physiol.

[42] Waters AM, Beales PL (2011). Ciliopathies: an expanding disease spectrum. Pediatr Nephrol.

[43] Murcia NS, Richards WG, Yoder BK, Mucenski ML, Dunlap JR, Woychik RP (2000). The Oak Ridge Polycystic Kidney (orpk) disease gene is required for left-right axis determination. Development.

[44] Lin F, Hiesberger T, Cordes K, Sinclair AM, Goldstein LSB, Somlo S, Igarashi P (2003). Kidney-specific inactivation of the KIF3A subunit of kinesin-II inhibits renal ciliogenesis and produces polycystic kidney disease. Proc Natl Acad Sci USA.

[45] Yoder BK, Hou X, Guay-Woodford LM (2002). The polycystic kidney disease proteins, polycystin-1, polycystin-2, polaris, and cystin, are co-localized in renal cilia. J Am Soc Nephrol.

[46] Pereira RC, Gitomer BY, Chonchol M, Harris PC, Noche KJ, Salusky IB, Albrecht L (2021). V Characterization of primary cilia in osteoblasts isolated from patients with ADPKD and CKD. JBMR Plus.

[47] Lim J, Li X, Yuan X, Yang S, Han L, Yang S. Primary cilia control cell alignment and patterning in bone development via ceramide-PKCζ-β-catenin signaling. Commun Biol. 2020;3. 10.1038/s42003-020-0767-x. 10.1038/s42003-020-0767-x PMC6985158 31988398

[48] Delous M, Baala L, Salomon R, Laclef C, Vierkotten J, Tory K, Golzio C, Lacoste T, Besse L, Ozilou C (2007). The ciliary gene RPGRIP1L is mutated in cerebello-oculo-renal syndrome (Joubert syndrome type B) and Meckel syndrome. Nat Genet.

[49] Perrault I, Saunier S, Hanein S, Filhol E, Bizet AA, Collins F, Salih MAM, Gerber S, Delphin N, Bigot K (2012). Mainzer-Saldino syndrome is a ciliopathy caused by IFT140 mutations. Am J Hum Genet.

[50] Mortellaro C, Bello L, Pucci A, Lucchina AG, Migliario M (2010). Saldino-Mainzer syndrome: nephronophthisis, retinitis pigmentosa, and cone-shaped epiphyses. J Craniofac Surg.

[51] Turco AE, Padovani EM, Chiaffoni GP, Peissel B, Rossetti S, Marcolongo A, Gammaro L, Maschio G, Pignatti PF (1993). Molecular genetic diagnosis of autosomal dominant polycystic kidney disease in a newborn with bilateral cystic kidneys detected prenatally and multiple skeletal malformations. J Med Genet.

[52] Behmel A, Plöchl E, Rosenkranz W (1984). A new X-linked dysplasia gigantism syndrome: identical with the Simpson dysplasia syndrome?. Hum Genet.

[53] Gorlin RJ, Psaume J (1962). Orodigitofacial dysostosis—a new syndrome: a study of 22 cases. J Pediatr.

[54] Feather SA, Winyard PJ, Dodd S, Woolf AS (1997). Oral-facial-digital syndrome type 1 is another dominant polycystic kidney disease: clinical, radiological and histopathological features of a new kindred. Nephrol Dial Transplant Off Publ Eur Dial Transpl Assoc Ren Assoc.

[55] Beales PL, Bland E, Tobin JL, Bacchelli C, Tuysuz B, Hill J, Rix S, Pearson CG, Kai M, Hartley J (2007). IFT80, which encodes a conserved intraflagellar transport protein, is mutated in Jeune asphyxiating thoracic dystrophy. Nat Genet.

[56] Ruiz-Perez VL, Ide SE, Strom TM, Lorenz B, Wilson D, Woods K, King L, Francomano C, Freisinger P, Spranger S (2000). Mutations in a new gene in Ellis-van Creveld syndrome and Weyers acrodental dysostosis. Nat Genet.

[57] Robins DG, French TA, Chakera TM (1976). Juvenile nephronophthisis associated with skeletal abnormalities and hepatic fibrosis. Arch Dis Child.

[58] Popović-Rolović M, Calić-Perisíc N, Bunjevacki G, Negovanović D (1976). Juvenile nephronophthisis associated with retinal pigmentary dystrophy, cerebellar ataxia, and skeletal abnormalities. Arch Dis Child.

[59] Chen C-P (2007). Meckel syndrome: genetics, perinatal findings, and differential diagnosis. Taiwan J Obstet Gynecol.

[60] Lauweryns B, Leys A, Van Haesendonck E, Missotten L (1993). Senior-Løken syndrome with marbelized fundus and unusual skeletal abnormalities. Graefes Arch Clin Exp Ophthalmol.

[61] Wassermann F, Yaeger JA (1965). Fine structure of the osteocyte capsule and of the wall of the lacunae in bone. Z Zellforsch Mik Anat.

[62] Tobin JL, Di Franco M, Eichers E, May-Simera H, Garcia M, Yan J, Quinlan R, Justice MJ, Hennekam RC, Briscoe J (2008). Inhibition of neural crest migration underlies craniofacial dysmorphology and Hirschsprung’s disease in Bardet-Biedl syndrome. Proc Natl Acad Sci USA.

[63] Marshall JD, Bronson RT, Collin GB, Nordstrom AD, Maffei P, Paisey RB, Carey C, MacDermott S, Russell-Eggitt I, Shea SE (2005). New Alström syndrome phenotypes based on the evaluation of 182 cases. Arch Intern Med.

[64] Roth KE, Rieder CL, Bowser SS (1988). Flexible-substratum technique for viewing cells from the side: some in vivo properties of primary (9+ 0) cilia in cultured kidney epithelia. J Cell Sci.

[65] Praetorius HA, Spring KR (2001). Bending the MDCK cell primary cilium increases intracellular calcium. J Membr Biol.

[66] Tonna EA, Lampen NM (1972). Electron microscopy of aging skeletal cells. I. Centrioles and solitary cilia. J Gerontol.

[67] Whitfield JF (2003). Primary cilium - is it an osteocyte’s strain-sensing flowmeter?. J Cell Biochem.

[68] Whitfield JF (2008). The solitary (primary) cilium-a mechanosensory toggle switch in bone and cartilage cells. Cell Signal.

[69] Coughlin TR, Voisin M, Schaffler MB, Niebur GL, McNamara LM (2015). Primary cilia exist in a small fraction of cells in trabecular bone and marrow. Calcif Tissue Int.

[70] Nauli SM, Alenghat FJ, Luo Y, Williams E, Vassilev P, Li X, Elia AEH, Lu W, Brown EM, Quinn SJ (2003). Polycystins 1 and 2 mediate mechanosensation in the primary cilium of kidney cells. Nat Genet.

[71] Hoey DA, Chen JC, Jacobs CR. The primary cilium as a novel extracellular sensor in bone. Front Endocrinol (Lausanne). 2012;3. 10.3389/fendo.2012.00075. 10.3389/fendo.2012.00075 PMC3374377 22707948

[72] Espinha LC, Hoey DA, Fernandes PR, Rodrigues HC, Jacobs CR (2014). Oscillatory fluid flow influences primary cilia and microtubule mechanics. Cytoskeleton.

[73] Hoey DA, Downs ME, Jacobs CR (2012). The mechanics of the primary cilium: an intricate structure with complex function. J Biomech.

[74] Hoey DA, Tormey S, Ramcharan S, O’Brien FJ, Jacobs CR (2012). Primary cilia-mediated mechanotransduction in human mesenchymal stem cells. Stem Cells.

[75] Corrigan MA, Johnson GP, Stavenschi E, Riffault M, Labour M-N, Hoey DA (2018). TRPV4-mediates oscillatory fluid shear mechanotransduction in mesenchymal stem cells in part via the primary cilium. Sci Rep.

[76] Johnson GP, Fair S, Hoey DA (2021). Primary cilium-mediated MSC mechanotransduction is dependent on Gpr161 regulation of hedgehog signalling. Bone.

[77] Hoey DA, Kelly DJ, Jacobs CR (2011). A role for the primary cilium in paracrine signaling between mechanically stimulated osteocytes and mesenchymal stem cells. Biochem Biophys Res Commun.

[78] Young Y-N, Downs M, Jacobs CR (2012). Dynamics of the primary cilium in shear flow. Biophys J.

[79] Downs ME, Nguyen AM, Herzog FA, Hoey DA, Jacobs CR (2014). An experimental and computational analysis of primary cilia deflection under fluid flow. Comput Methods Biomech Biomed Engin.

[80] McGlashan SR, Jensen CG, Poole CA (2006). Localization of extracellular matrix receptors on the chondrocyte primary cilium. J Histochem Cytochem.

[81] Reilly GC, Haut TR, Yellowley CE, Donahue HJ, Jacobs CR (2003). Fluid flow induced PGE2 release by bone cells is reduced by glycocalyx degradation whereas calcium signals are not. Biorheology.

[82] Marszalek JR, Ruiz-Lozano P, Roberts E, Chien KR, Goldstein LSB (1999). Situs inversus and embryonic ciliary morphogenesis defects in mouse mutants lacking the KIF3A subunit of kinesin-II. Proc Natl Acad Sci USA.

[83] Temiyasathit S, Tang WJ, Leucht P, Anderson CT, Monica SD, Castillo AB, Helms JA, Stearns T, Jacobs CR (2012). Mechanosensing by the primary cilium: deletion of Kif3a reduces bone formation due to loading. PLoS ONE.

[84] Qiu N, Xiao Z, Cao L, Buechel MM, David V, Roan E, Quarles LD (2012). Disruption of Kif3a in osteoblasts results in defective bone formation and osteopenia. J Cell Sci.

[85] Lee KL, Guevarra MD, Nguyen AM, Chua MC, Wang Y, Jacobs CR (2015). The primary cilium functions as a mechanical and calcium signaling nexus. Cilia.

[86] Martín-Guerrero E, Tirado-Cabrera I, Buendía I, Alonso V, Gortázar AR, Ardura JA (2020). Primary cilia mediate parathyroid hormone receptor type 1 osteogenic actions in osteocytes and osteoblasts via Gli activation. J Cell Physiol.

[87] Xu H, Guan Y, Wu J, Zhang J, Duan J, An L, Shang P (2014). Polycystin 2 is involved in the nitric oxide production in responding to oscillating fluid shear in MLO-Y4 cells. J Biomech.

[88] Xiao Z, Dallas M, Qiu N, Nicolella D, Cao L, Johnson M, Bonewald L, Quarles LD (2011). Conditional deletion of Pkd1 in osteocytes disrupts skeletal mechanosensing in mice. FASEB J.

[89] Kwon RY, Temiyasathit S, Tummala P, Quah CC, Jacobs CR (2010). Primary cilium-dependent mechanosensing is mediated by adenylyl cyclase 6 and cyclic AMP in bone cells. FASEB J.

[90] Riffault M, Johnson GP, Owen MM, Javaheri B, Pitsillides AA, Hoey DA (2020). Loss of adenylyl cyclase 6 in leptin receptor-expressing stromal cells attenuates loading-induced endosteal bone formation. JBMR Plus.

[91] Duffy MP, Sup ME, Guo XE (2021). Adenylyl cyclase 3 regulates osteocyte mechanotransduction and primary cilium. Biochem Biophys Res Commun.

[92] Spasic M, Jacobs CR (2017). Lengthening primary cilia enhances cellular mechanosensitivity. Eur Cells Mater.

[93] Wang P, Tang C, Wu J, Yang Y, Yan Z, Liu X, Shao X, Zhai M, Gao J, Liang S (2019). Pulsed electromagnetic fields regulate osteocyte apoptosis, RANKL/OPG expression, and its control of osteoclastogenesis depending on the presence of primary cilia. J Cell Physiol.

[94] Hao X, Wang D, Yan Z, Ding Y, Zhang J, Liu J, Shao X, Liu X, Wang L, Luo E (2023). Bone deterioration in response to chronic high-altitude hypoxia is attenuated by a pulsed electromagnetic field via the primary cilium/HIF-1α axis. J Bone Miner Res.

[95] Ding D, Yang X, Luan H-Q, Wu X-T, Sun L-W, Fan Y-B (2020). The microgravity induces the ciliary shortening and an increased ratio of anterograde/retrograde intraflagellar transport of osteocytes. Biochem Biophys Res Commun.

[96] Kathem SH, Mohieldin AM, Abdul-Majeed S, Ismail SH, Altaei QH, Alshimmari IK, Alsaidi MM, Khammas H, Nauli AM, Joe B (2014). Ciliotherapy: a novel intervention in polycystic kidney disease. J Geriatr Cardiol JGC.

[97] Upadhyay VS, Muntean BS, Kathem SH, Hwang JJ, AbouAlaiwi WA, Nauli SM (2014). Roles of dopamine receptor on chemosensory and mechanosensory primary cilia in renal epithelial cells. Front Physiol.

[98] Spasic M, Duffy MP, Jacobs CR. Fenoldopam sensitizes primary cilia-mediated mechanosensing to promote osteogenic intercellular signaling and whole bone adaptation. J Bone Miner Res. 2022;37:972–82. 10.1002/jbmr.4536. 10.1002/jbmr.4536 PMC9098671 35230705

[99] Ding D, Yang X, Luan H-Q, Wu X-T, He C, Sun L-W, Fan Y-B (2020). Pharmacological regulation of primary cilium formation affects the mechanosensitivity of osteocytes. Calcif Tissue Int.

[100] Köttgen M, Buchholz B, Garcia-Gonzalez MA, Kotsis F, Fu X, Doerken M, Boehlke C, Steffl D, Tauber R, Wegierski T (2008). TRPP2 and TRPV4 form a polymodal sensory channel complex. J Cell Biol.

[101] Truong ME, Bilekova S, Choksi SP, Li W, Bugaj LJ, Xu K, Reiter JF (2021). Vertebrate cells differentially interpret ciliary and extraciliary cAMP. Cell.

[102] Goff E, Cohen A, Shane E, Recker RR, Kuhn G, Müller R (2022). Large-scale osteocyte lacunar morphological analysis of transiliac bone in normal and osteoporotic premenopausal women. Bone.

[103] McNamara LM (2021). Osteocytes and estrogen deficiency. Curr Osteoporos Rep.

[104] Windahl SH, Saxon L, Börjesson AE, Lagerquist MK, Frenkel B, Henning P, Lerner UH, Galea GL, Meakin LB, Engdahl C (2013). Estrogen receptor-α is required for the osteogenic response to mechanical loading in a ligand-independent manner involving its activation function 1 but not 2. J Bone Miner Res.

[105] Hagino H, Raab DM, Kimmel DB, Akhter MP, Recker RR (1993). Effect of ovariectomy on bone response to in vivo external loading. J Bone Miner Res.

[106] Sharma D, Ciani C, Marin PAR, Levy JD, Doty SB, Fritton SP (2012). Alterations in the osteocyte lacunar–canalicular microenvironment due to estrogen deficiency. Bone.

[107] Verbruggen SW, Vaughan TJ, McNamara LM (2016). Mechanisms of osteocyte stimulation in osteoporosis. J Mech Behav Biomed Mater.

[108] Deepak V, Kayastha P, McNamara LM (2017). Estrogen deficiency attenuates fluid flow-induced [Ca2+]i oscillations and mechanoresponsiveness of MLO-Y4 osteocytes. FASEB J.

[109] Li H, Li R-X, Wan Z-M, Xu C, Li J-Y, Hao Q-X, Guo Y, Liu L, Zhang X-Z (2013). Counter-effect of constrained dynamic loading on osteoporosis in ovariectomized mice. J Biomech.

[110] Sutton MM, Duffy MP, Verbruggen SW, Jacobs CR. Osteoclastogenesis requires primary cilia disassembly and can be inhibited by promoting primary cilia formation pharmacologically. Cells Tissues Organs. 2023;1. 10.1159/000531098. 10.1159/000531098 PMC10863750 37231815

[111] Geoghegan IP, McNamara LM, Hoey DA. Estrogen withdrawal alters cytoskeletal and primary ciliary dynamics resulting in increased Hedgehog and osteoclastogenic paracrine signalling in osteocytes. Sci Rep. 2021;11. 10.1038/s41598-021-88633-6. 10.1038/s41598-021-88633-6 PMC8085225 33927279

